# Adherence to the Mediterranean diet and incident cardiovascular outcomes among older adults

**DOI:** 10.3389/fnut.2026.1926159

**Published:** 2026-08-19

**Authors:** Emanuele Marzetti, Riccardo Calvani, Isabel Rodriguez-Sanchez, Samuel da Silva Aguiar, Anna Picca, Matteo Tosato, Stefano Cacciatore, Francesco Landi, Helio José Coelho Júnior

**Affiliations:** 1 Fondazione Policlinico Universitario Agostino Gemelli IRCCS, Rome, Italy; 2 Department of Geriatrics, Orthopedics and Rheumatology, Università Cattolica Del Sacro Cuore, Rome, Italy; 3 Department of Geriatrics, Hospital Universitario Clínico San Carlos, Madrid, Spain; 4 Fundación para la Investigación Biomédica del Hospital Clínico San Carlos (IdISSC), Madrid, Spain; 5 Postgraduate Program in Physical Education, Catholic University of Brasília, Brasília, Brazil; 6 Center for Proteomic and Biochemical Analysis, Postgraduate Program in Genomic Sciences and Biotechnology, Catholic University of Brasília, Brasília, Brazil; 7 Department of Medicine and Surgery, LUM University, Casamassima, Italy; 8 Department of Physiology and Aging, College of Medicine, University of Florida, Gainesville, FL, United States

**Keywords:** diet pattern, heart attack, hypertension, malnutrition, myocardial infarction, stroke

## Abstract

**Aim:**

The present study examined the prospective associations between adherence to the Mediterranean (MED) diet, its individual components, and incident cardiovascular disease (CVD) among community-dwelling older adults.

**Methods:**

Data were obtained from the English Longitudinal Study of Ageing (ELSA). Participants aged ≥65 years without hypertension at baseline (Wave 9, 2018–2019) were followed through Wave 11 (2023–2024). Adherence to the MED diet was assessed using the Medi-Lite score. Incident hypertension, myocardial infarction, and stroke were identified through self-reported physician diagnoses. Logistic regression analyses were performed to examine the prospective associations between MED diet adherence, individual dietary components, and cardiovascular outcomes, adjusting for age, sex, and caloric intake.

**Results:**

A total of 338 participants (74.6 ± 6.2 years; 56.6% women) were included. During follow-up, the incidence of hypertension, myocardial infarction, and stroke was 12.4%, 2.6%, and 2.6%, respectively. No statistically significant associations were detected between overall MED diet adherence and incident hypertension (OR = 0.992, 95% CI = 0.586–1.679), myocardial infarction (OR = 1.422, 95% CI = 0.381–5.302), or stroke (OR = 0.808, 95% CI = 0.360–1.811). Likewise, none of the individual MED diet components was significantly associated with incident CVD outcomes.

**Conclusion:**

In conclusion, no statistically significant associations were detected between adherence to the MED diet and incident CVD among community-dwelling older adults in England. However, given the relatively low number of cardiovascular events, particularly myocardial infarction and stroke, these findings should be interpreted with caution. Further studies with longer follow-up periods and larger numbers of CVD events are warranted to provide more precise estimates and further clarify these associations.

## 1 Introduction

The Mediterranean (MED) diet is a dietary pattern traditionally observed in countries bordering the MED Sea and reflects a combination of historical, environmental, and cultural influences that have evolved over generations (1, 2). Its main characteristics include a predominance of plant-based foods, such as fruits, vegetables, legumes, whole grains, nuts, and olive oil, together with moderate consumption of fish and seafood. In contrast, red and processed meat are consumed sparingly, while alcohol intake, when present, is generally moderate and often accompanies meals (1, 2). Interest in this dietary pattern grew substantially following evidence from large epidemiological studies conducted in the late twentieth century, which suggested that individuals with greater adherence to MED eating habits experienced lower mortality rates (3, 4). Subsequent research has expanded these findings, demonstrating associations between the MED diet and a wide range of favorable health outcomes (5–10).

High adherence to the MED diet has long been associated with improved cardiovascular health (11–13). These benefits are thought to arise from multiple interacting mechanisms, including increased intake of bioactive compounds and micronutrients, attenuation of chronic low-grade inflammation and oxidative stress, and improved endothelial function through enhanced nitric oxide bioavailability (14, 15). Collectively, these effects favorably influence established cardiovascular risk markers, such as blood pressure and serum cholesterol levels (14, 15).

These results are particularly important for older adults, as cardiovascular (CVD) remains one of the leading causes of morbidity and mortality in this population (16). Nevertheless, evidence specifically focusing on older adults remains limited, particularly in non-MED settings. Although previous studies have investigated the associations between individual dietary components and cardiovascular outcomes, few have simultaneously examined both overall adherence to the MED diet and its individual components in relation to incident CVD among community-dwelling older adults. Addressing this gap may help clarify whether the observed benefits are attributable to the dietary pattern as a whole or to specific components.

Therefore, the present study aims to investigate the associations of both overall adherence to the MED diet and its individual components with the incidence of CVD, including hypertension, myocardial infarction, and stroke, among community-dwelling older adults.

## 2 Materials and methods

### 2.1 Study design and participants

Data were obtained from the English Longitudinal Study of Ageing (ELSA), an ongoing population-based cohort study involving adults aged 50 years and older living in England. ELSA includes repeated assessments covering physical function, cognitive performance, lifestyle behaviors, chronic diseases, and biological measures (17). Participant recruitment began in 2002–2003, and the latest available assessment corresponded to Wave 11 (2023–2024). Detailed dietary information was first incorporated in Wave 9 (2018–2019), which was therefore considered the baseline for the present investigation. Follow-up analyses were based on data from Wave 11, allowing approximately 6 years of observation. All procedures were conducted according to the Declaration of Helsinki principles.

The analytical sample was limited to participants aged ≥65 years who reported not having received a diagnosis of hypertension at baseline (variable = hedawbp). Individuals using mobility devices (manual wheelchairs, electric wheelchairs, scooters, or mobility buggies) were excluded because they generally represent a population with substantial functional dependence, a high burden of multimorbidity, and complex healthcare needs, all of which may independently influence dietary habits, nutritional requirements, and cardiovascular risk. Participants with major neurological, psychiatric, or oncological diseases were also excluded because these conditions frequently require disease-specific dietary management and may substantially alter nutritional intake, metabolism, physical function, and cardiovascular risk, thereby increasing clinical heterogeneity and the potential for residual confounding. To improve data reliability, participants presenting implausible dietary reports were removed from the analysis. Extreme caloric intake values (<500 or >4,600 kcal/day) and unrealistic protein intake values (<12 or >200 g/day) were considered physiologically incompatible with habitual intake in free-living older adults. Ethical approval for ELSA was granted by the National Research Ethics Service (MREC/01/2/91), and reporting followed the STROBE recommendations (18).

### 2.2 Dietary assessment and adherence to the Mediterranean

Dietary intake was evaluated using the Food Frequency Questionnaire (FFQ) applied during ELSA Wave 9 through the Oxford web-based dietary assessment platform. The instrument estimates habitual food and beverage intake over the preceding 12 months by recording consumption frequency across a wide range of food items. Reported frequencies were converted into estimated daily intake using standard portion sizes provided in the ELSA nutritional database. Nutrient composition values were derived from UK food composition tables linked to the FFQ.

Adherence to the MED diet was quantified using the Medi-Lite score (0–16) (19, 20). The score incorporates intake frequency of core MED dietary components, including vegetables, fruits, legumes, cereals, fish, olive oil, and moderate alcohol consumption. Greater intake of these foods contributed positively to the score, whereas red and processed meats and high-fat dairy products were reverse scored. Individual component scores were summed, with higher values reflecting stronger adherence to the MED dietary pattern.

### 2.3 Cardiovascular diseases

Incidence of CVD was defined based on self-reported new physician diagnoses since the previous interview, including hypertension (variable = hediia1), heart attack (including, myocardial infarction or coronary thrombosis; variable = hediia3), and stroke (variable = hediia7).

### 2.4 Covariates

Age and sex were assessed at baseline. Caloric intake was expressed as mean daily energy intake (kcal/day).

### 2.5 Statistical analysis

Continuous variables are presented as mean ± standard deviation, whereas categorical variables are expressed as frequency and percentage. Prospective analyses evaluated whether baseline dietary variables predicted clinical outcomes at follow-up. Logistic regression models were applied for dichotomous outcomes. Adjusted logistic regression models included age, sex, and caloric intake. These covariates were selected *a priori* based on their established associations with both dietary patterns and cardiovascular disease. A parsimonious adjustment strategy was adopted to minimize the risk of model overfitting and unstable estimates given the limited number of myocardial infarction and stroke events, in accordance with recommendations for multivariable logistic regression modelling in studies with relatively few outcome events (21, 22). Physical activity and smoking status were reported as baseline characteristics to describe the study population but were not included in the primary adjustment models. Findings were interpreted according to odds ratios (OR) and corresponding 95% confidence intervals. Statistical significance was established at *p* < 0.05. All analyses were performed using SPSS version 23.

## 3 Results

### 3.1 Participants’ characteristics

The main characteristics of study participants of the present study are shown in Table 1 (*n* = 338). Participants were relatively younger older adults (mean age = 74.6 years) with adequate caloric intake (mean caloric intake = 2122.7 kcal) according to their weight. The incidence of hypertension, heart attack, and stroke was 12.4, 2.6, and 2.6%, respectively.

**Table 1:** Baseline characteristics of the study participants (Wave 9; *n* = 338).

Variables	Values
Age, y	74.6 ± 6.2
Female, %	56.6
Weight, kg	73.5 ± 15.8
Hospitalized during COVID-19, %	7
Current smoking, %	27.8
Caloric intake, kcal/day	2122.7 ± 731.2
MED diet, mean scores	9.2 ± 1.8
Physical activity, MET-minutes per day	561.2 ± 377.5

COVID, coronavirus disease 2019; MED, Mediterranean diet; MET, metabolic equivalent of task.

### 3.2 Associations between Mediterranean diet and cardiovascular diseases

Results of the unadjusted and adjusted logistic regression analyses examining the associations between adherence to the MED diet and incident CVD outcomes are presented in Table 2. No statistically significant associations were detected between the MED diet score and the incidence of hypertension, myocardial infarction, or stroke in either the unadjusted or adjusted analyses.

**Table 2:** Regression analyses of the associations between the Mediterranean diet score and cardiovascular disease (*n* = 338).

	Unadjusted			Adjusted		
	OR	95% CI	*p*-value	OR	95% CI	*p*-value
Hypertension	1.102	0.696, 1.745	0.680	0.992	0.586, 1.679	0.976
Heart attack	1.204	0.446, 3.247	0.714	1.422	0.381, 5.302	0.600
Stroke	0.942	0.473, 1.877	0.865	0.808	0.360, 1.811	0.605

CI, confidence interval; OR, odds ratio.

Adjusted for age, sex, and caloric intake.

### 3.3 Associations between components of the Mediterranean diet and cardiovascular diseases

Results of the unadjusted and adjusted analyses examining the associations between individual MED diet components and incident CVD outcomes are presented in Table 3. No statistically significant associations were detected between any individual MED diet component and the incidence of hypertension, myocardial infarction, or stroke.

**Table 3:** Regression analyses of the associations between the Mediterranean diet components and cardiovascular disease (*n* = 338).

	Hypertension						Heart attack						Stroke					
	Unadjusted			Adjusted			Unadjusted			Adjusted			Unadjusted			Adjusted		
	OR	95% CI	*p*-value	OR	95% CI	*p*-value	OR	95% CI	*p*-value	OR	95% CI	*p*-value	OR	95% CI	*p*-value	OR	95% CI	*p*-value
Fruits	1.372	0.417, 4.514	0.603	1.275	0.352, 4.619	0.711	—	—	—	—	—	—	0.732	0.140, 3.817	0.711	0.788	0.134, 4.621	0.792
Vegetables	1.843	0.535, 6.346	0.332	1.529	0.420, 5.561	0.519	0.924	0.086, 9.898	0.948	1.135	0.071, 18.245	0.929	0.922	0.168, 5.050	0.925	0.642	0.110, 3.757	0.623
Legumes	0.815	0.298, 2.229	0.690	0.603	0.123, 2.963	0.534	—	—	—	—	—	—	—	—	—	—	—	—
Cereals and Grains	—	—	—	—	—	—	—	—	—	—	—	—	0.305	0.048, 1.945	0.209	—	—	—
Fish	0.768	0.272, 2.170	0.619	0.574	0.120, 2.740	0.486	0.579	0.110, 3.051	0.519	0.313	0.019, 5.087	0.414	—	—	—	—	—	—
Meat	0.509	0.070, 3.690	0.504	0.397	0.040, 3.894	0.427	—	—	—	—	—	—	—	—	—	—	—	—
Dairy products	0.502	0.088, 2.856	0.437	0.406	0.057, 2.913	0.370	1.573	0.306, 8.084	0.587	2.48	0.150, 41.033	0.526	—	—	—	—	—	—
Olive oil	3.833	0.546, 26.892	0.176	2.463	0.302, 20.119	0.400	—	—	—	—	—	—	2.182	0.125, 38.182	0.593	2.784	0.037, 20.591	0.642
Alcohol	—	—	—	—	—	—	—	—	—	—	—	—	—	—	—	—	—	—

CI, confidence interval; OR, odds ratio.

Adjusted for age, sex, and caloric intake.

“—” indicates that the corresponding analysis was not performed because of insufficient observations to produce reliable estimates.

## 4 Discussion

The present study investigated the prospective associations between adherence to the MED diet and incident CVD among normotensive community-dwelling older adults. No statistically significant associations were detected between overall MED diet adherence or its individual components and the incidence of hypertension, heart attack, or stroke during follow-up. Although these findings differ from those reported in several previous studies, they should be interpreted with caution. In particular, the relatively low number of CVD events, especially heart attack and stroke, reduced the precision of the estimated associations and may have limited the statistical power to detect modest effects.

Numerous studies have investigated the associations between adherence to the MED diet and the occurrence of CVD. Cowell et al. (11) reported that higher adherence to the MED diet was associated with a 13% lower risk of hypertension in a cohort of nearly 60,000 adults. Similarly, Rosato et al. (12) observed lower risks of heart attack and stroke among individuals with greater adherence to the MED diet, and these associations remained significant after adjustment for geographic region (MED versus non-MED countries). However, evidence in older adults has been less consistent. Although similar findings have been reported in this population (13), the associations were no longer statistically significant when analyses were restricted to individuals aged ≥70 years. Furthermore, substantial heterogeneity has been identified across studies.

Taken together, the present findings suggest that no statistically significant association between adherence to the MED diet and incident CVD was detected in this cohort of community-dwelling older adults. However, these findings should be interpreted cautiously and should not be considered evidence that the MED diet is ineffective for CVD prevention, particularly given the relatively low number of CVD events. Although several biological mechanisms have been proposed to explain the cardioprotective effects of the MED diet, including attenuation of inflammation, oxidative stress, and endothelial dysfunction (23–26), these pathways were not evaluated in the present study and therefore should be considered as possible explanations rather than direct interpretations of our findings.

These findings are also consistent with the observation that none of the individual components of the MED diet was significantly associated with incident CVD. It is possible that the cardiovascular benefits attributed to the MED diet result from the synergistic interaction among its components rather than from the effects of individual food groups alone. Moreover, many components of the MED diet are shared with other healthy dietary patterns, such as the Dietary Approaches to Stop Hypertension (DASH), Nordic Diet, and WHO-diet index, and several of these components have individually been associated with improved cardiovascular outcomes. Therefore, the absence of significant associations for individual MED diet components should be interpreted with caution and does not necessarily preclude a potential benefit of the overall dietary pattern.

Previous studies have reported significant associations between several individual components of the MED diet and cardiovascular health. For example, higher consumption of fruits and vegetables has been associated with a lower risk of hypertension and CVD (27–29), while greater whole grain intake has been linked to a reduced risk of CHD and incident CVD (30). Similar associations have also been reported for olive oil (31) and moderate wine consumption (32). Although these findings appear to differ from those of the present study, variations in study populations, dietary assessment methods, outcome definitions, and follow-up duration may partly explain these discrepancies. Furthermore, the relatively low number of CVD events in the present cohort may have limited our ability to detect modest associations for individual MED diet components.

However, sensitivity analyses from previous studies suggest that not all individual foods within each MED diet component contribute equally to cardiovascular health. For example, several commonly consumed vegetables (e.g., potatoes, legumes, eggplant, and zucchini), fruits (e.g., bananas, oranges, and berries), and whole-grain products were not consistently associated with lower CVD risk (27, 29, 30). Therefore, the absence of statistically significant associations for the overall MED diet components observed in the present study may partly reflect the heterogeneous effects of the individual foods comprising each component. Future studies examining the contribution of specific foods within each MED diet component may help clarify their individual roles in CVD prevention.

Another possible explanation for the absence of statistically significant associations relates to factors that were not evaluated in the present study. Previous research has suggested that the cardioprotective effects of the MED diet may be attenuated among individuals with multimorbidity or at risk of malnutrition (26). Both conditions may influence dietary habits, nutritional requirements, and cardiovascular risk, thereby potentially modifying the association between adherence to the MED diet and incident CVD. However, because these factors were not assessed in the present analyses, their potential contribution remains speculative and warrants further investigation.

Therefore, it is possible that unmeasured factors, including multimorbidity and nutritional status, contributed to the absence of statistically significant associations observed in the present study. In addition, the MED diet score used in this study was based on cut-offs derived from previous literature rather than being specifically developed for the UK population, which may have introduced some degree of exposure misclassification. However, because multimorbidity and nutritional status were not included in the adjusted analyses, largely due to the limited number of CVD events and the need to avoid model overfitting, these hypotheses remain speculative and should be investigated in future studies with larger samples and a greater number of outcome events.

An alternative or complementary explanation is that the present study was conducted in a non-MED population. Whether the MED diet can be directly translated to non-MED countries remains a matter of debate (33). Differences in food availability, food composition, culinary practices, and dietary habits may influence adherence to the MED diet and the health effects associated with its individual components (33). For example, the primary sources of monounsaturated fats, methods of vegetable preparation, patterns of alcohol consumption, and the types of meat and dairy products consumed often differ substantially between MED and non-MED populations (33). Consequently, these contextual differences may have contributed to the absence of statistically significant associations observed in the present study. Future studies comparing MED and non-MED populations using harmonized dietary assessment methods may help clarify this issue.

The present study has several limitations. First, participants from the ELSA cohort represented a relatively healthy population of community-dwelling older adults, characterized by a low prevalence of mobility limitations, preserved walking speed, and generally adequate nutritional intake. Consequently, our findings may not be generalizable to older adults with greater functional impairment, institutionalized populations, or individuals with complex chronic conditions. Second, follow-up blood pressure measurements were not available in the ELSA dataset, precluding the assessment of longitudinal changes in blood pressure. Third, participants included in ELSA Wave 11 (2023–2024) were survivors of the COVID-19 pandemic, which may have introduced survivor bias. Fourth, nutritional data were available for only a subset of eligible participants, potentially limiting the representativeness of the analytical sample. Finally, the incidence of CVD events, particularly heart attack and stroke, was low, likely reflecting both the relatively healthy profile of the cohort and the duration of follow-up. Consequently, although the number of incident hypertension events was sufficient to support the adjusted analyses, the relatively small number of heart attack and stroke events reduced the precision of the estimated associations, resulting in wide confidence intervals for several estimates and limiting the statistical power to detect modest associations. In addition, the limited number of events required a parsimonious adjustment strategy to minimize model overfitting, restricting the inclusion of additional covariates and leaving the possibility of residual confounding from non-included cardiovascular risk factors. Therefore, the absence of statistically significant associations should not be interpreted as evidence of the absence of an association. Future studies with larger samples, longer follow-up periods, and a greater number of CVD events are warranted to provide more precise estimates and further clarify these associations.

In conclusion, no statistically significant associations were detected between adherence to the MED diet and incident cardiovascular events (i.e., hypertension, myocardial infarction, and stroke) among normotensive community-dwelling older adults. Likewise, no statistically significant associations were detected for the individual components of the MED diet. However, these findings should be interpreted with caution given the relatively low number of cardiovascular events, particularly myocardial infarction and stroke, which resulted in limited statistical power and wide confidence intervals for several estimates. Therefore, the absence of statistically significant associations should not be interpreted as evidence of the absence of an association. Future studies with larger samples, longer follow-up periods, and greater numbers of cardiovascular events are warranted to provide more precise estimates and further clarify the relationship between the MED diet and cardiovascular health. In addition, studies examining specific foods within each MED diet component (e.g., different types of fruits), accounting for nutritional status and multimorbidity, and comparing MED and non-MED populations may provide further insight into these associations.

## Data Availability

The datasets presented in this study can be found in online repositories. The names of the repository/repositories and accession number(s) can be found at: https://www.elsa-project.ac.uk/.
